# GelMA Versus Agarose Hydrogels in Pancreatic Cancer 3D Spheroid Modeling: Effects on Morphology, HIF-1α Expression, and Gemcitabine Response

**DOI:** 10.3390/gels12050377

**Published:** 2026-04-30

**Authors:** Zeynep Celik, Hatice Gumushan Aktas, Bulent Aktas, Serife Yalcin

**Affiliations:** 1 Biology Department, Faculty of Arts & Science, Harran University, Sanliurfa 63050, Türkiye; haticeaktas@harran.edu.tr; 2 Mechanical Engineering Department, Faculty of Engineering, Harran University, Sanliurfa 63050, Türkiye; 3 Physics Department, Faculty of Arts & Science, Harran University, Sanliurfa 63050, Türkiye; serifeyalcin@harran.edu.tr

**Keywords:** hydrogel microenvironment, GelMA hydrogel, agarose hydrogel, 3D tumor spheroids, hypoxia (HIF-1α)

## Abstract

Given its highly aggressive nature and poor clinical outcome, pancreatic ductal adenocarcinoma (PDAC) requires physiologically relevant in vitro models that more accurately reflect tumor biology and drug response. In this study, adhesive and non-adhesive hydrogel microenvironments were comparatively evaluated for pancreatic cancer spheroid modeling using PANC-1 and MIA PaCa-2 cells. Gelatin methacryloyl (GelMA) hydrogels were synthesized, photocrosslinked, and optimized in terms of stability, swelling, degradation, and cytocompatibility, while 3% agarose was used as a non-adhesive counterpart. Although the optimized GelMA formulation showed adequate structural stability and no cytotoxicity, it did not support spheroid formation. In contrast, agarose enabled the formation of compact, viable, and proliferative spheroids in both cell lines. Agarose-derived spheroids exhibited time-dependent growth, positive Ki-67 staining, and increased HIF-1α expression under 3D conditions, indicating the establishment of hypoxia-associated tumor-like microenvironments. Gemcitabine treatment induced a time-dependent reduction in spheroid viability, while viable cell populations persisted throughout exposure, reflecting the heterogeneous therapeutic response typical of 3D tumor models. Overall, these findings provide a comparative, microenvironment-based assessment of pancreatic cancer spheroid modeling, indicating that hydrogel-dependent differences in adhesivity and structural dynamics are important determinants of spheroid assembly, hypoxia-associated molecular adaptation, and chemotherapeutic response. Overall, these findings provide a comparative, microenvironment-based assessment of pancreatic cancer spheroid modeling, indicating that hydrogel-dependent differences in adhesivity and structural dynamics are important determinants of spheroid assembly, hypoxia-associated molecular adaptation, and chemotherapeutic response. Overall, these findings provide a comparative, microenvironment-based assessment of pancreatic cancer spheroid modeling, indicating that hydrogel-dependent differences in adhesivity and structural dynamics are important determinants of spheroid assembly, hypoxia-associated molecular adaptation, and chemotherapeutic response.

## 1. Introduction

Pancreatic ductal adenocarcinoma (PDAC) is one of the most aggressive and lethal malignancies, characterized by late diagnosis, intrinsic chemoresistance, and a remarkably poor prognosis. According to global epidemiological data, pancreatic cancer accounts for approximately 2.5% of all newly diagnosed cancer cases worldwide but is responsible for nearly 4.5% of cancer-related deaths, reflecting its disproportionately high mortality rate [1]. Despite significant advances in oncology research, survival outcomes for PDAC have shown only marginal improvement over recent decades, in stark contrast to the declining mortality rates observed for many other solid tumors. Consequently, pancreatic cancer currently ranks as the fourth leading cause of cancer-related mortality and is projected to become the second leading cause if effective therapeutic strategies are not developed [2].

Elucidating the biological mechanisms underlying pancreatic cancer progression, therapeutic resistance, and tumor–microenvironment interactions relies heavily on experimental model systems. Conventional two-dimensional (2D) monolayer cultures remain widely used because of their simplicity and reproducibility, but they do not adequately reproduce the three-dimensional architecture, cell–cell/cell–matrix interactions, and diffusion gradients that shape tumor behavior in vivo [3,4,5,6,7,8,9].

Accordingly, three-dimensional (3D) culture systems have emerged as more physiologically relevant platforms that better mimic tumor organization and microenvironmental constraints [10,11,12,13,14,15]. Among these, spheroid-based models are particularly valuable because they reproduce multicellular aggregation, diffusion-limited oxygen and nutrient gradients, and treatment-response patterns that are difficult to capture in 2D systems [4,13]. These models can be generated either by scaffold-free approaches or by biomaterial-based platforms, including hydrogels and other non-adhesive substrates that modulate cell–cell and cell–matrix interactions [16,17,18].

Agarose and gelatin methacryloyl (GelMA) are among the most widely employed biomaterials in 3D culture systems, yet they generate fundamentally distinct microenvironments that can strongly influence tumor architecture and function. Agarose provides a non-adhesive environment that favors cell–cell aggregation, whereas GelMA is a photocrosslinkable, cell-adhesive matrix that supports cell attachment and matrix remodeling [19]. Recent studies have shown that the hydrogel microenvironment strongly influences spheroid compaction, matrix remodeling, and therapeutic response in pancreatic cancer models, while 3D pancreatic spheroids display microenvironment-dependent phenotypic heterogeneity that cannot be reproduced in 2D culture [20,21]. Moreover, both natural and synthetic hydrogels, including GelMA-based systems, are increasingly recognized as key regulators of tumor architecture, mechanosensing, and drug response [22,23]. Despite their broad use, their comparative suitability for pancreatic cancer spheroid modeling remains insufficiently defined, particularly with respect to spheroid formation, hypoxia-associated signaling, and drug response.

Accordingly, the present study addresses a specific gap in PDAC 3D culture research: while adhesive and non-adhesive biomaterial systems have been broadly discussed in the literature, the direct comparative impact of GelMA and agarose on pancreatic cancer spheroid formation and downstream tumor-relevant behavior under identical conditions has not been systematically clarified. What remained unclear was not simply whether these matrices differ in adhesivity, but how those differences translate into spheroid morphology, structural maturation, hypoxia-associated HIF-1α signaling, and gemcitabine response in the same PDAC experimental framework. In this study, we therefore performed a matched side-by-side evaluation of GelMA and agarose using PANC-1 and MIA PaCa-2 cells, integrating hydrogel physicochemical characterization with biological readouts. This approach provides a more precise basis for selecting hydrogel microenvironments in pancreatic cancer spheroid modeling and for linking material properties to functionally relevant tumor-like outcomes.

## 2. Results and Discussion

The present study shows that hydrogel choice strongly influences pancreatic cancer spheroid behavior. Although both GelMA and agarose are widely used in 3D culture, they created distinct microenvironments and led to markedly different outcomes in spheroid formation, structural maturation, hypoxia-associated signaling, and gemcitabine response. The following sections relate these biological differences to the physicochemical and mechanical properties of the two hydrogel systems.

### 2.1. Optimization of GelMA Photocrosslinking and Hydrogel Stability

Gelatin methacryloyl (GelMA) hydrogels were synthesized in-house and systematically optimized using lithium phenyl-2,4,6-trimethylbenzoylphosphinate (LAP) as a photoinitiator under 405 nm irradiation. GelMA formulations at 5% and 10% (*w*/*v*) combined with 0.05% or 0.1% LAP were evaluated under three photocuring conditions (0 s, 15 s, and 45 s).

**Figure 1:**
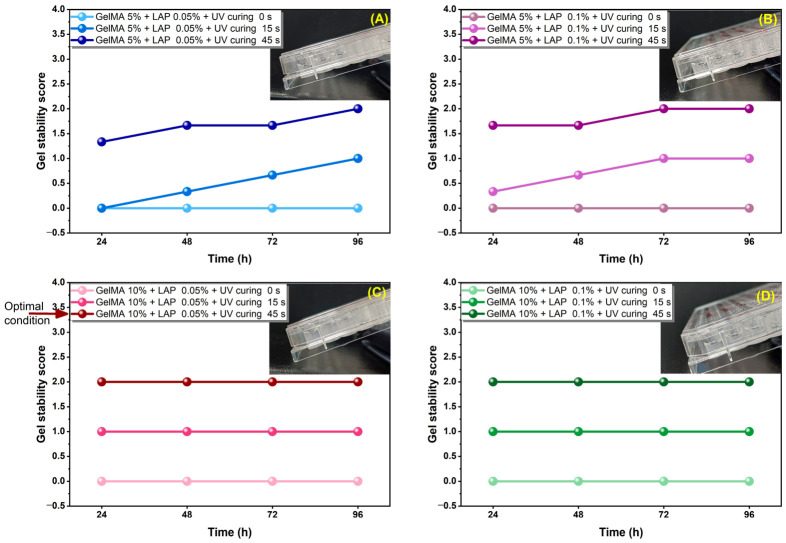
Effect of GelMA concentration, LAP content, and UV curing duration on hydrogel stability over time. Semi-quantitative stability profiles of GelMA hydrogels prepared at different formulations [(**A**) GelMA 5% + LAP 0.05%, (**B**) GelMA 5% + LAP 0.1%, (**C**) GelMA 10% + LAP 0.05%, and (**D**) GelMA 10% + LAP 0.1%] and exposed to different UV curing times (0, 15, and 45 s) were monitored over 24–96 h. Hydrogel stability was assessed using a macroscopic scoring system (0 = liquid, 1 = partially stable gel, 2 = stable gel). Representative images of the corresponding hydrogel samples are included in each panel. Among the tested conditions, GelMA 10% + LAP 0.05% cured for 45 s exhibited the highest structural stability throughout the entire observation period and was therefore selected for subsequent biological experiments.

Hydrogel stability was assessed semi-quantitatively by monitoring structural integrity in PBS at 37 °C over 96 h (Figure 1). Non-crosslinked gels rapidly lost structural integrity, whereas photocrosslinked constructs exhibited markedly improved stability. Among all formulations, GelMA 10% + 0.05% LAP cured for 45 s demonstrated the most consistent structural preservation, maintaining a stable hydrogel morphology throughout the observation period. Increasing the LAP concentration to 0.1% did not significantly improve macroscopic stability, suggesting that a lower photoinitiator loading was sufficient for network formation.

These findings confirm that proper photocrosslinking is essential for GelMA stability and exclude hydrogel dissolution as the primary reason for the absence of spheroid formation in subsequent experiments. Instead, the results suggest that the intrinsic cell–matrix interactions in GelMA may regulate cell aggregation behavior.

### 2.2. FT-IR and ^1^H-NMR Analysis Results

**Figure 2:**
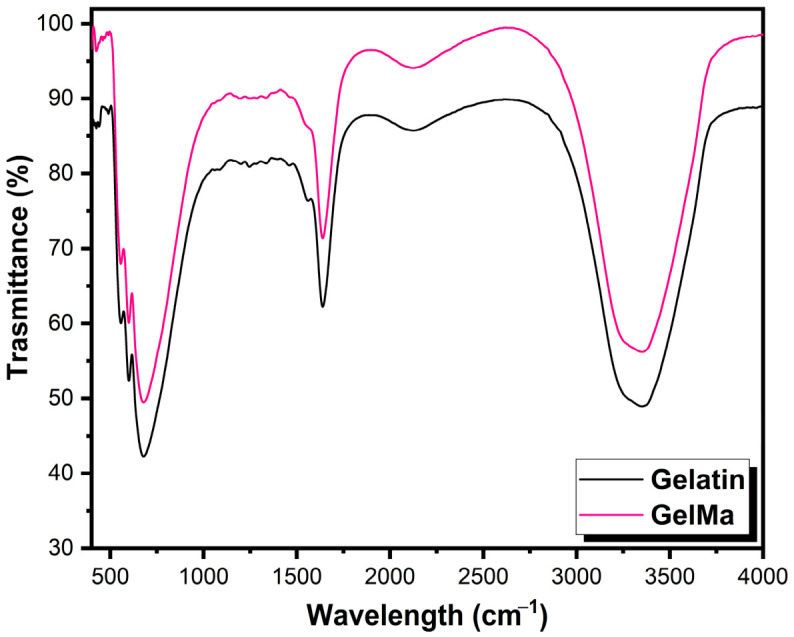
The FT-IR spectra of Gelatin and GelMA.

FT-IR measurements were conducted to identify the biomolecules and bonds responsible for the structural and functional stabilization of gelatin and GelMA. The characteristic peaks observed for both gelatin and GelMA are presented in Figure 2. The overall similarity of the spectra confirms that GelMA retains the fundamental protein backbone structure of gelatin. However, critical evidence of successful methacrylation is observed in the modification of lysine groups. Notably, the intensity of the peak around 1557 cm^−1^, associated with the N–H bending of primary amines in gelatin, significantly decreased or shifted following the reaction. This change confirms that the primary amino groups of gelatin were successfully reacted with methacrylic anhydride to form methacrylamide linkages. Proteins consist of amino acids linked by amide bonds. Among these, the amide I and II bands are the most prominent vibrational modes of the protein backbone. The repeating units of polypeptides and proteins generate nine characteristic infrared (IR) absorption bands, which include amide A, B, and I–VII. These amide bands correspond to various vibrational modes of the peptide bond [24,25]. The peak at 3448 cm^−1^ is attributed to the presence of water molecules involved in hydrogen bonding, as well as amide-A. The peak at 1641 cm^−1^ corresponds to amide-I, which is associated with C=O stretching vibrations of the peptide linkages. While the Amide-II band is still observed at 1553 cm^−1^ due to the preserved peptide backbone, the subtle decrease in the adjacent 1557 cm^−1^ region serves as the chemical indicator of GelMA synthesis. The band at 1242 cm^−1^ is linked to amide-III, which arises from the in-plane vibrations of the C–N and N–H groups of the bound amide. The peaks between 1457 cm^−1^ and 1334 cm^−1^ are attributed to the symmetric and asymmetric bending vibrations of the methyl group. Amide IV, at 677 cm^−1^, corresponds to OCN bending, while amide VI, at 593 cm^−1^, is linked to out-of-plane C=O bending [24].

**Figure 3:**
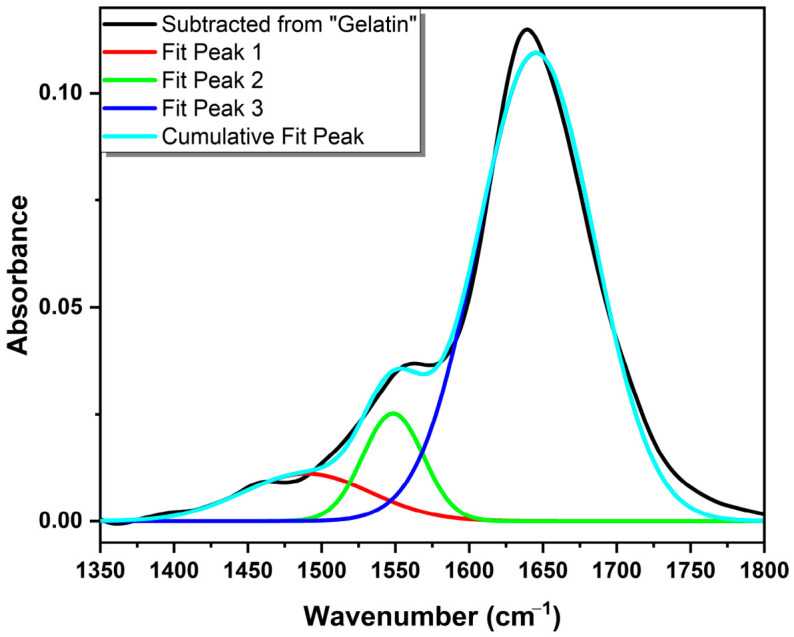
FT-IR peak deconvolutions of Gelatin.

The FT-IR spectra in the 1400–1800 cm^−1^ region have been analyzed using peak deconvolution of gelatin and GelMA, and the deconvolution graphs are shown in Figure 3. The individual sub-peaks were fitted using a Gaussian function to accurately resolve the Amide I and Amide II bands. The fitting process was performed until a high correlation coefficient was achieved, ensuring the cumulative fit represents the raw data. The high degree of fit between the deconvolution peaks and experimental data is evidenced by low Reduced Chi-Sqr values of 0.00548 for Gelatin and ≈0.00527 for GelMA. While the protein’s essential framework is preserved, the deconvolution reveals subtle shifts in the Amide I and II sub-peak distributions. Specifically, the conversion of primary amines into methacrylamide groups leads to a reorganization of the hydrogen bonding network, reflected in the relative area changes in the sub-peaks. Unlike generic protein descriptions, our data specifically shows that the methacrylation process induces a localized reorganization of the amide environments without causing significant denaturation of the gelatin precursor. This system-specific analysis confirms that the chemical modification is successfully targeted to the functional side chains while maintaining the structural integrity of the protein backbone. These findings collectively demonstrate that while the methacrylation process is clearly evidenced by sub-peak shifts, the overall FT-IR profiles remain stable, confirming that the modification proceeds without fundamentally destabilizing the chemical framework of gelatin.

**Figure 4:**
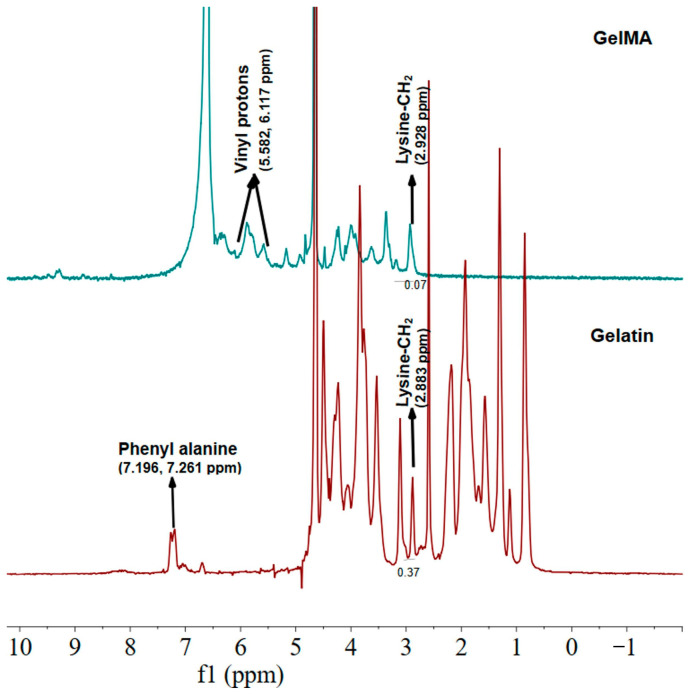
^1^H-NMR spectra of gelatin and GelMA samples.

Representative ^1^H-NMR spectra of gelatin and GelMA samples are shown in Figure 4. The ^1^H-NMR spectra clearly confirmed the successful methacrylation of gelatin. In the GelMA spectrum, characteristic methacrylate vinyl proton peaks appeared at approximately 5.6–6.0 ppm, while the intensity of the lysine methylene signal (~2.9 ppm) decreased compared to native gelatin, indicating the consumption of primary amine groups during functionalization. Based on peak integral analysis, the degree of methacrylation (DoM) was determined to be approximately 81%, indicating a high level of functionalization. Such a high DoM is known to enhance photocrosslinking efficiency and increase network density, leading to improved mechanical stability, as widely reported in previous GelMA studies [19,26,27]. However, increasing methacrylation also reduces the number of free amine groups while preserving cell-interactive motifs such as RGD sequences, thereby strengthening cell–matrix interactions [26,28]. This balance between high crosslinking capacity and retained bioactivity likely explains the observed behavior in the present study, where GelMA formed a structurally stable yet highly adhesive microenvironment that promoted cell spreading rather than cell–cell aggregation, ultimately inhibiting spheroid formation. Similar observations have been reported in the literature, where highly methacrylated GelMA hydrogels favor cell attachment and matrix remodeling but can limit the formation of compact multicellular spheroids [26,28].

### 2.3. Swelling Behavior and Degradation Characteristics

The swelling and degradation behaviors of the optimized GelMA hydrogel (10% GelMA + 0.05% LAP, 45 s UV curing) and 3% agarose hydrogel were systematically investigated to elucidate their structure property relationships and their implications for 3D cell culture applications.

#### 2.3.1. Swelling Behavior: Network Architecture and Water Uptake Dynamics

**Figure 5:**
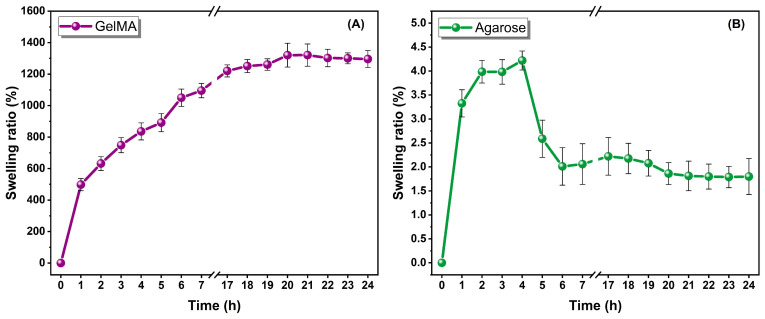
Swelling behavior of hydrogel samples: (**A**) GelMA and (**B**) agarose.

As presented in Figure 5, GelMA hydrogels exhibited a rapid and extensive swelling response, reaching approximately 500% within the first hour and exceeding 1200–1300% at equilibrium. The swelling profile showed a biphasic pattern consisting of an initial rapid water uptake phase followed by a plateau, indicating equilibrium swelling. This behavior is characteristic of gelatin-derived hydrogels, in which hydrophilic amino acid residues and a relatively low effective crosslink density enable significant water absorption and network expansion [19,26]. From a polymer physics perspective, the high swelling ratio of GelMA can be attributed to hydrophilic polymer backbone (gelatin-derived chains), elastic network expansion governed by osmotic pressure, and moderate crosslink density even after photocuring. These features result in increased mesh size (ξ), which enhances water diffusion and solute transport. However, excessive swelling is also associated with reduced mechanical confinement and diminished cell–cell interaction.

In contrast, agarose hydrogels demonstrated minimal swelling (<30%), with rapid stabilization and negligible variation over time (Figure 5). Agarose forms a physically crosslinked double-helix network, resulting in high network density, small pore size, and restricted polymer chain mobility. As previously reported, increasing agarose concentration significantly reduces swelling due to tighter chain packing and decreased free volume [29,30].

#### 2.3.2. Degradation Behavior: Stability and Network Integrity

**Figure 6:**
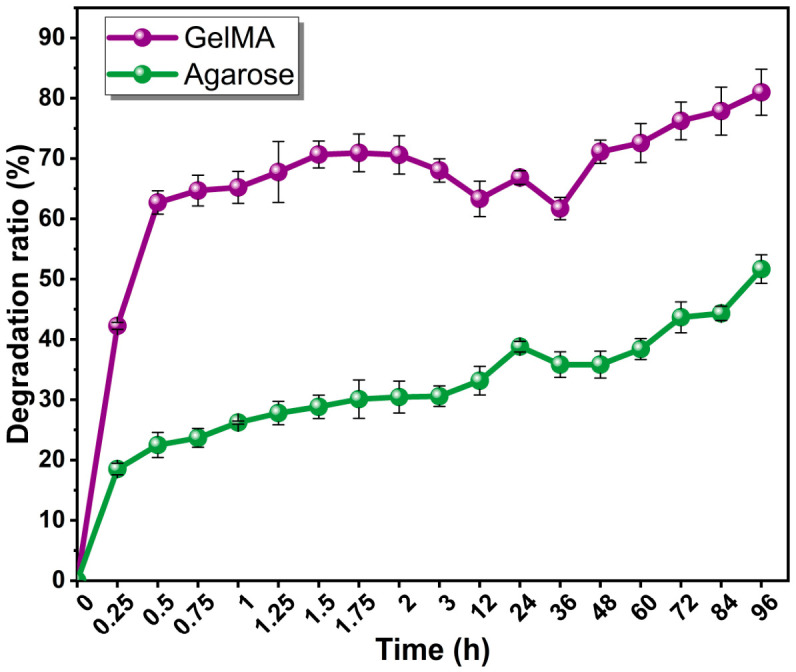
Degradation properties of GelMA and Agarose hydrogel samples.

As shown in Figure 6, the two hydrogel systems exhibited markedly different degradation profiles under physiological conditions. The optimized GelMA hydrogel showed a rapid initial mass loss, reaching approximately 40% degradation at the early stage and gradually increasing to nearly 70% before approaching a plateau, whereas agarose displayed a much slower and more limited degradation profile, remaining around 30% mass loss throughout the test period. This difference is consistent not only with the distinct chemical nature of the two matrices, but also with their network organization. In the present study, GelMA exhibited a substantially lower effective network density than agarose (ν_eff_ ≈ 4.55 mol m^−3^ vs. 11.48 mol m^−3^), which, indicating a looser and less mechanically confining network. Together with its high swelling behavior, this lower network density likely facilitated greater water penetration, enhanced polymer-chain mobility, and accelerated structural relaxation, thereby contributing to the faster mass loss observed for GelMA. By contrast, the higher effective network density of agarose is consistent with its dense physically crosslinked double-helix structure, which restricts chain mobility, limits water-induced network loosening, and helps preserve structural integrity over time [26,27,29,30,31]. From a biological perspective, these degradation characteristics are highly relevant because matrix stability directly affects the persistence of the 3D microenvironment during culture. The rapid degradation of GelMA suggests the formation of a more dynamic and permissive matrix, whereas the slower degradation of agarose indicates a more stable and persistent scaffold. When considered together with the swelling and mechanical data, Figure 6 therefore supports the interpretation that agarose preserved a more compact and structurally durable network, while GelMA formed a highly hydrated, low-density matrix that was more susceptible to degradation and less capable of maintaining the physical confinement required for stable spheroid formation [8,32].

### 2.4. Hydrogel Stiffness and Textural Properties

**Figure 7:**
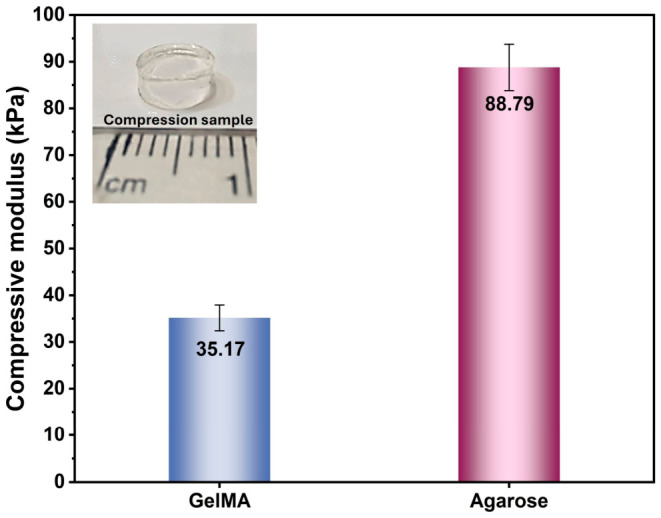
Compressive Young’s modulus of GelMA and agarose hydrogels.

As shown in Figure 7, agarose hydrogels exhibited a markedly higher compressive Young’s modulus than GelMA hydrogels (88.79 vs. 35.17 kPa), indicating a substantially stiffer load-bearing network under uniaxial compression. This trend was further supported by the texture profile analysis results, in which agarose showed a much higher hardness value than GelMA (7.01 vs. 1.65 N). Together, these findings indicate that agarose forms a mechanically stronger and more deformation-resistant matrix, whereas GelMA behaves as a softer hydrogel with lower resistance to compressive loading. This mechanical contrast is consistent with previous reports showing that GelMA hydrogels typically display moduli in the tens-of-kPa range depending on polymer concentration, curing conditions, and processing parameters, while 3% agarose can reach substantially higher stiffness because of its dense, physically crosslinked network [26,27,29,31]. For example, a tuned GelMA system with a Young’s modulus of 32.6 ± 1.9 kPa has been reported in the literature, which is very close to the GelMA value observed here [33], whereas 3.0% agarose hydrogels have been reported to exhibit a Young’s modulus of 81.1 ± 2.1 kPa, closely matching the stiffness range measured in the present study [34].

The higher stiffness of agarose can be attributed to its well-known thermoreversible double-helix aggregation and the formation of a tightly packed three-dimensional network with limited chain mobility [29,31]. Classical studies on agarose mechanics have shown that increasing agarose concentration decreases mesh size and increases gel rigidity, which explains why the 3% agarose used here generated a much stronger structure than GelMA [29]. In contrast, GelMA contains a highly hydrated gelatin-derived backbone and forms a more compliant network in which the final mechanical response depends strongly on photocrosslinking density, formulation parameters, and thermal history [26,27,33]. This interpretation is also fully consistent with the swelling data presented earlier in the manuscript, where GelMA showed extensive water uptake, while agarose exhibited minimal swelling [26,27,29,30,31]. In other words, the lower swelling and denser network architecture of agarose are directly reflected in its higher compressive modulus and hardness [29,31].

Comparative texture profile analysis (TPA) results for GelMA and agarose hydrogels are presented in Table 1. Interestingly, the TPA parameters also reveal that stiffness alone does not fully characterize the hydrogel’s mechanical behavior. Although agarose was much harder, its cohesiveness (0.30) was lower than that of GelMA (1.00), while the springiness values of both hydrogels were nearly identical (~1.0). This suggests that both materials were able to recover their shape after deformation to a similar extent, but they differed in how well their internal structures tolerated repeated compression. The lower cohesiveness of agarose may reflect a more brittle or fracture-prone response during the second compression cycle, which is consistent with the behavior of rigid physically crosslinked gels [29,31]. By contrast, the high cohesiveness of GelMA indicates that, despite being softer, its network retained structural continuity more effectively under repeated loading. A similar concentration-dependent increase in hardness and springiness has also been reported for agar gels in texture profile analysis studies, supporting the interpretation that stronger agarose networks display firmer but less compliant textural behavior [35].

Another important point is that the “adhesiveness” measured by TPA should not be confused with biological cell-adhesiveness. The zero adhesiveness recorded for GelMA in the texture test only indicates negligible work of detachment between the probe and the hydrogel surface during instrumental compression. It does not contradict the known cell-interactive character of GelMA, which arises from its gelatin-derived biochemical motifs [26,27,28]. Therefore, from a biological perspective, the present mechanical data suggest a meaningful structure-function relationship: agarose provides a stiffer and more mechanically confining environment, while GelMA provides a softer and more deformable matrix. When considered together with the swelling, degradation, and spheroid formation results, these data support the conclusion that the stiff, low-swelling, non-adhesive agarose network is more favorable for preserving a mechanically stable environment that promotes cell–cell aggregation, whereas the softer GelMA matrix is more permissive and less mechanically restrictive, which may contribute to the lack of compact spheroid formation under the tested conditions [8,26,28,32].

### 2.5. Cytotoxicity Assessment of GelMA Extracts

**Figure 8:**
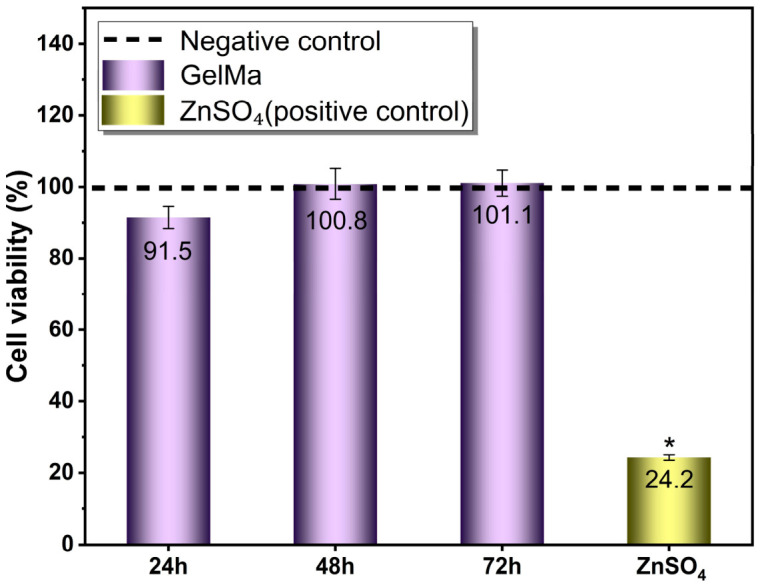
Cytotoxicity evaluation of GelMA extracts using MEM elution assay on NIH/3T3 fibroblast cells. Cell viability was assessed after 24, 48, and 72 h using the Alamar Blue assay. The dashed line represents the negative control (100% viability). GelMA-treated groups showed no significant cytotoxicity compared to the control (*p* > 0.05). (*) The positive control (2 mM ZnSO_4_) exhibited significantly reduced cell viability (*p* < 0.001). Data are presented as mean ± SE.

The cytotoxicity of GelMA hydrogel, which was determined to have optimum stability (GelMA 10% + LAP 0.05% + UV curing 45 s), was evaluated using a MEM elution assay, and the results are shown in Figure 8. Cell viability values for NIH/3T3 fibroblast cells were 91.5%, 100.8%, and 101.1% at 24, 48, and 72 h, respectively. No statistically significant difference was observed between the GelMA-treated groups and the negative control (*p* > 0.05), indicating that the GelMA extracts did not induce cytotoxicity.

According to ISO 10993-5 guidelines, materials are considered non-cytotoxic when cell viability exceeds 70% [36,37,38]. All GelMA-treated samples remained well above this threshold, confirming the excellent cytocompatibility of the optimized hydrogel formulation. In contrast, the positive control (2 mM ZnSO_4_) resulted in a significant reduction in cell viability to 24.2%, which was statistically different from both the negative control and GelMA-treated groups (*p* < 0.001). This confirms the sensitivity and validity of the assay.

The slight increase in metabolic activity observed at 48 and 72 h may be attributed to enhanced cellular proliferation or favorable interactions with bioactive components released from the GelMA matrix. These findings are consistent with previous reports demonstrating that GelMA hydrogels support cell viability and proliferation due to their ECM-like composition [26,27].

### 2.6. Spheroid Formation, Morphological Characterization, and Hypoxia-Associated HIF-1α Expression

**Figure 9:**
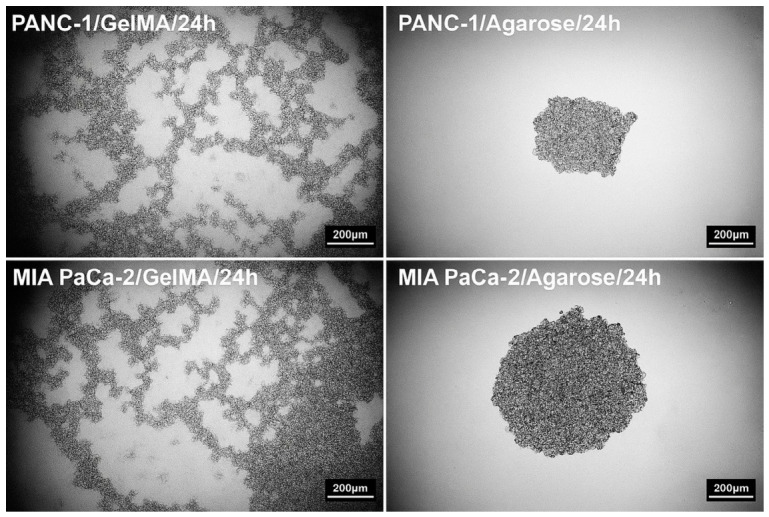
Spheroid formation behavior of pancreatic cancer cell lines in GelMA and agarose hydrogels. PANC-1 and MIA PaCa-2 cells were cultured under identical conditions in optimized GelMA hydrogels and 3% agarose matrices. No spheroid formation was observed in GelMA, whereas compact and well-defined spheroids were successfully formed in agarose. These findings highlight the influence of hydrogel properties on cell aggregation and spheroid formation. Phase-contrast microscope, magnification 4×.

Importantly, the differences observed at the material level were not confined to hydrogel characterization but translated directly into distinct biological outcomes. GelMA created a hydrated, bioactive, and adhesive microenvironment that promoted cell–matrix interactions, whereas agarose provided a confined, non-adhesive setting that favored cell–cell cohesion. This contrast proved decisive for pancreatic cancer spheroid development. Accordingly, spheroid formation and related cellular responses were systematically examined in optimized GelMA and 3% agarose hydrogels under identical experimental conditions. Although the selected GelMA formulation exhibited adequate structural stability and cytocompatibility, it did not support spheroid formation. In contrast, both PANC-1 and MIA PaCa-2 cells generated compact and well-defined spheroids in 3% agarose hydrogels (Figure 9).

The inability of cells to form spheroids in GelMA can be attributed to its bioactive and cell-adhesive nature, primarily due to the presence of arginine-glycine-aspartic acid (RGD) motifs, which promote strong cell–matrix interactions. This adhesive environment favors cell spreading rather than aggregation, thereby limiting the cell–cell interactions required for spheroid compaction. Similar observations have been reported in previous studies demonstrating that adhesive hydrogels can inhibit spheroid formation despite supporting cell viability [8,26]. In contrast, agarose provides a non-adhesive, structurally stable microenvironment that promotes cell–cell interactions and spontaneous aggregation, consistent with previous reports indicating that non-adhesive hydrogels are highly effective for generating uniform tumor spheroids [16,32]. Importantly, this observation should be interpreted within the formulation window examined in the present study rather than as a general limitation of GelMA as a biomaterial platform. The ^1^H-NMR analysis confirmed a relatively high degree of methacrylation (DoM ≈ 81%), indicating substantial photocrosslinking potential; however, DoM reflects the availability of reactive methacrylate groups rather than the absolute final crosslink density of the cured hydrogel. Consistent with this, the mechanical data showed that the tested GelMA formulation formed a markedly softer network than agarose, with a lower compressive modulus and lower apparent/effective network density $\left(\nu_{e,eff}\right)$ estimated from modulus-based analysis (approximately 4.55 mol m^−3^ for GelMA versus 11.48 mol m^−3^ for agarose). Together with the swelling and degradation results, these findings suggest that the GelMA formulation used here generated a highly hydrated, compliant, and cell-interactive microenvironment that favored cell–matrix interactions over stable cell–cell aggregation. Therefore, the present results indicate that the optimized GelMA condition used in this study did not support compact spheroid formation under the tested conditions, while alternative GelMA formulations with different stiffness, ligand presentation, or crosslinking degree may yield different outcomes.

**Figure 10:**
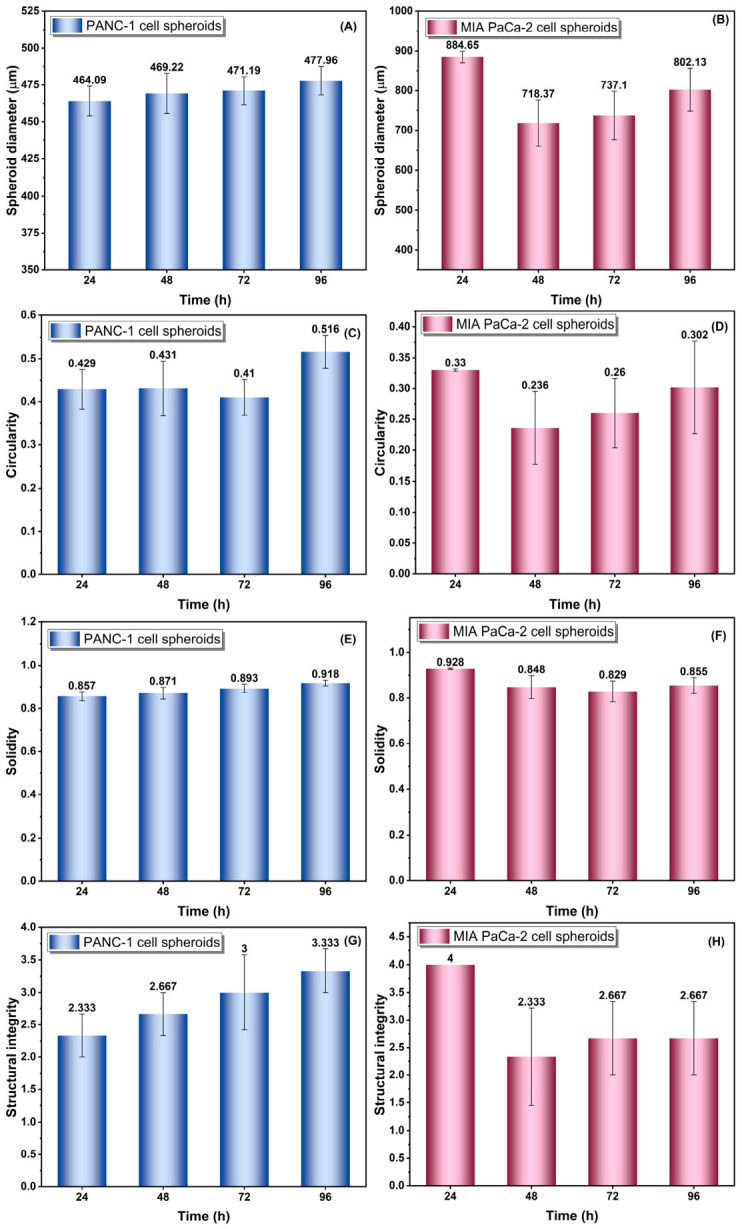
Time-dependent quantitative characterization of PANC-1 and MIA PaCa-2 spheroids cultured in 3% agarose hydrogels: (**A**,**B**) Changes in spheroid diameter; (**C**,**D**) circularity; (**E**,**F**) solidity; and (**G**,**H**) structural integrity at 24, 48, 72, and 96 h. Panels (**A**,**C**,**E**,**G**) correspond to PANC-1 spheroids, whereas panels (**B**,**D**,**F**,**H**) correspond to MIA PaCa-2 spheroids. Data are presented as mean ± standard error (*n* ≥ 7). No statistically significant differences were observed among the time points for spheroid diameter within each cell line (*p* > 0.05).

Figure 10 demonstrates that spheroid maturation in agarose was both time-dependent and cell-line-specific. PANC-1 spheroids showed a gradual increase in diameter, together with improved circularity, solidity, and structural integrity, indicating progressive compaction, surface regularization, and structural stabilization over time. Consistent with these findings, aspect ratio analysis also supported the observed differences in spheroid morphology and is provided in the Supplementary Information (Figure S1). In contrast, MIA PaCa-2 spheroids exhibited an initially larger size followed by a marked reduction at 48 h and only partial recovery thereafter, accompanied by lower circularity and solidity values, suggesting an early compaction–reorganization phase and a more heterogeneous architecture. Such transient decreases in spheroid size during early maturation have been reported previously and are generally attributed to the reassembly of loosely associated cells into denser multicellular structures [16,39]. The more regular and progressively stabilized morphology of PANC-1 spheroids, compared with the more dynamic restructuring behavior of MIA PaCa-2 spheroids, is also consistent with earlier pancreatic cancer spheroid studies showing cell-line-dependent differences in aggregation kinetics and 3D structural organization [40,41,42]. Importantly, both spheroid models remained within a size range compatible with diffusion-limited oxygen and nutrient transport, supporting their physiological relevance as tumor-like 3D culture systems [32,39]. Although a time-dependent trend was observed in spheroid diameter, no statistically significant differences were detected among the 24, 48, 72, and 96 h time points within either cell line (*p* > 0.05).

**Figure 11:**
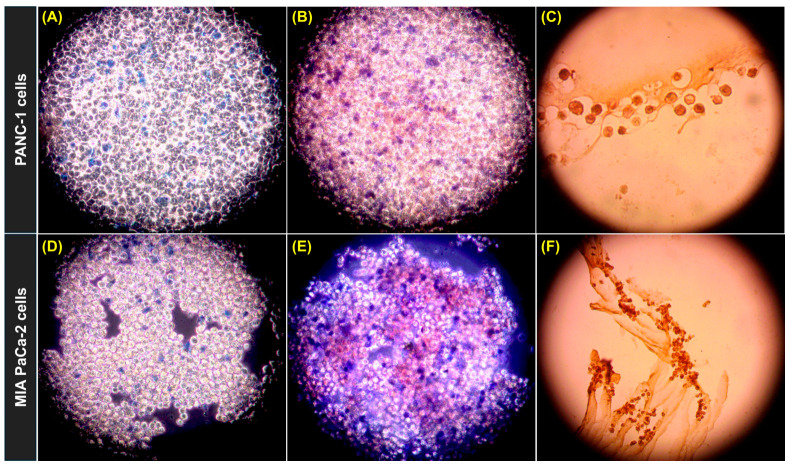
Morphological and viability characterization of spheroids formed in 3% agarose hydrogels. (**A**–**C**) PANC-1 spheroids; (**D**–**F**) MIA PaCa-2 spheroids. (**A**,**D**) Trypan blue staining showing non-viable cells (blue) and viable cells (unstained). (**B**,**E**) Combined trypan blue and neutral red staining indicates viable cells (red) and dead cells (blue). (**C**,**F**) Ki-67 immunohistochemical staining showing proliferating cells (brown), confirming active DNA synthesis within spheroids. Magnification 20×.

Morphological and staining analyses indicated that the formed spheroids were viable and biologically active. Trypan blue staining (Figure 11A,D) demonstrated minimal cell death, while neutral red staining (Figure 11B,E) confirmed active lysosomal function and metabolic activity. In addition, Ki-67 immunostaining (Figure 11C,F) showed the presence of DAB-positive nuclei within the spheroid structure, supporting the presence and spatial distribution of cells with proliferative activity. In the present study, Ki-67 staining should be interpreted as a supportive qualitative histological finding rather than a quantitative proliferation endpoint. Taken together, these observations indicate that agarose-based spheroids were not only structurally compact but also biologically active systems.

**Figure 12:**
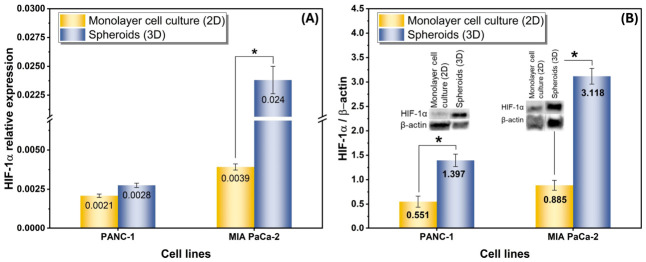
Comparison of HIF-1α expression in 2D monolayer and 3D spheroid pancreatic cancer models: (**A**) Relative HIF-1α mRNA expression levels in PANC-1 and MIA PaCa-2 cells cultured under 2D monolayer and 3D spheroid conditions, determined by quantitative real-time PCR and normalized to an internal reference gene, and (**B**) Relative HIF-1α protein expression under the same conditions, assessed by Western blotting and expressed as the HIF-1α/β-actin ratio. Representative immunoblot bands for HIF-1α and β-actin are shown in the insets. Data are presented as mean ± standard error. * *p* < 0.05 between the indicated groups.

Figure 12 confirms that the agarose-based 3D spheroid model generated a hypoxia-associated tumor-like microenvironment in both pancreatic cancer cell lines. At the transcript level (Figure 12A), HIF-1α expression increased in 3D relative to 2D culture in both models, but the magnitude of this increase was modest in PANC-1 and much more pronounced in MIA PaCa-2. This trend became even clearer at the protein level (Figure 12B), where the HIF-1α/β-actin ratio was markedly higher in 3D spheroids than in 2D monolayers in both cell lines, with MIA PaCa-2 again showing the strongest response. The stronger difference observed in Figure 10B than in Figure 10A is biologically meaningful, since HIF-1α is regulated predominantly through oxygen-dependent post-translational stabilization rather than through large transcriptional induction alone [39,43]. Thus, the combined qPCR and Western blot data indicate that spheroid architecture in agarose created diffusion-limited oxygen conditions sufficient to stabilize HIF-1α and activate a hypoxia-associated adaptive program. The more pronounced HIF-1α response in MIA PaCa-2 further suggests a greater hypoxia adaptation capacity in this cell line, which may reflect differences in spheroid organization, metabolic demand, or oxygen consumption, consistent with previous studies reporting cell-line-dependent hypoxia responses in pancreatic cancer spheroids [39,40,41,42]. From a tumor biology perspective, this finding is important because HIF-1α controls pathways involved in glycolysis, angiogenesis, survival, and treatment resistance [43]. Therefore, Figure 12 does not merely demonstrate a general difference between 2D and 3D culture; rather, it shows that the present 3D spheroid system reproduces a functionally relevant hypoxic phenotype at both the mRNA and protein levels, supporting its suitability for studying hypoxia-driven pancreatic cancer behavior and downstream therapeutic response. These findings support HIF-1α-associated hypoxic adaptation in a 3D global model, but additional markers are needed to characterize the complete downstream metabolic and drug resistance program. This observation is further supported by recent hydrogel-based spheroid studies, which show that non-adhesive or weakly interactive matrices promote multicellular aggregation, whereas adhesive hydrogels favor cell spreading and matrix interaction [44]. Similarly, advanced hydrogel platforms have been shown to regulate spheroid uniformity, structural organization, and drug response depending on matrix composition and architecture [45].

### 2.7. Gemcitabine Response and Hypoxia-Driven Drug Resistance in 3D Pancreatic Cancer Spheroids

**Figure 13:**
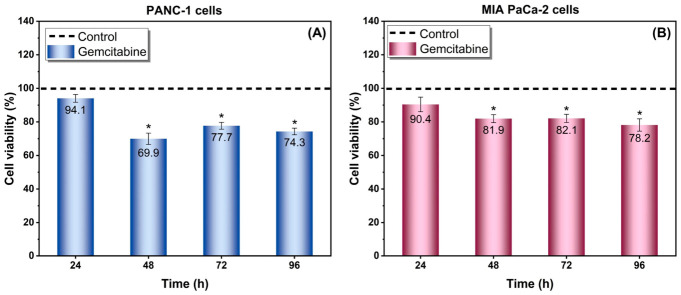
Time-dependent effect of gemcitabine on cell viability in 3D pancreatic cancer spheroids. (**A**) PANC-1 and (**B**) MIA PaCa-2 spheroids cultured in 3% agarose hydrogels were treated with gemcitabine for 24, 48, 72, and 96 h. Cell viability was assessed using the Alamar Blue assay and normalized to untreated controls. Data are presented as mean ± SE. A time-dependent reduction in viability was observed in both cell lines. *: Statistically significant difference between the control and the indicated test groups (*p* < 0.05).

For the 3D treatment experiments, gemcitabine was applied at the IC50-derived concentrations determined from preliminary 2D dose–response studies, namely 17.3 mM for PANC-1 and 20 mM for MIA PaCa-2. Gemcitabine treatment induced a time-dependent decrease in the viability of PANC-1 and MIA PaCa-2 spheroids grown in 3% agarose hydrogels, while both 3D models retained viable cell populations throughout the treatment period (Figure 13). This response pattern is consistent with the heterogeneous treatment behavior commonly reported in multicellular tumor spheroids [16,39].

**Figure 14:**
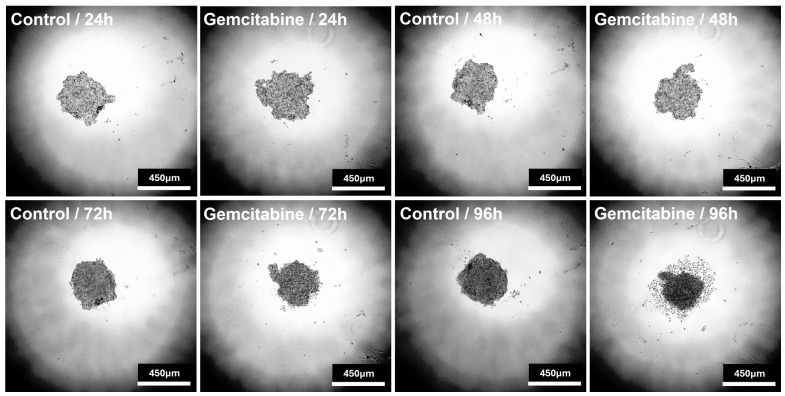
Gemcitabine response profile of PANC-1 spheroids. Cell viability of PANC-1 spheroids following gemcitabine treatment at different time points (24–96 h). A significant decrease in viability was observed at 48 h, followed by partial stabilization at later time points. Data are expressed as mean ± SE. Phase-contrast microscope, magnification 4×.

For PANC-1 spheroids, viability decreased from 94.1% at 24 h to 69.9% at 48 h, followed by a slight increase at 72 h and relative stabilization thereafter (Figure 13A and Figure 14). This pattern likely reflects the persistence of a treatment-tolerant cell fraction together with partial metabolic recovery of surviving cells within the spheroid, rather than complete reversal of gemcitabine activity. Since the Alamar Blue assay reflects metabolic activity rather than direct cell number, a modest rebound in signal may indicate adaptive recovery of viable residual cells. Such incomplete and non-monotonic responses are consistent with the heterogeneous behavior of multicellular tumor spheroids under chemotherapy [16,39,46].

**Figure 15:**
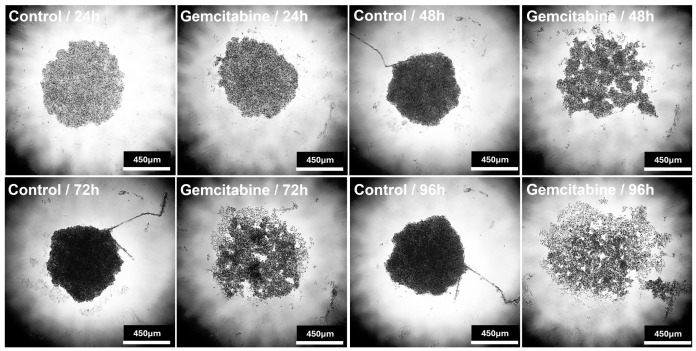
Gemcitabine response profile of MIA PaCa-2 spheroids. Cell viability of MIA PaCa-2 spheroids following gemcitabine treatment at 24, 48, 72, and 96 h. A moderate but sustained reduction in viability was observed over time. Data are presented as mean ± SE. Phase-contrast microscope, magnification 4×.

In MIA PaCa-2 spheroids, viability declined from 90.4% at 24 h to 81.9% at 48 h, remained at 82.1% at 72 h, and reached 78.2% at 96 h (Figure 13B and Figure 15). Compared with PANC-1, this more moderate but sustained decrease suggests a relatively more tolerant phenotype under 3D conditions. Such cell-line-dependent differences are compatible with prior reports showing that pancreatic cancer responses to gemcitabine are influenced by intrinsic resistance mechanisms, including differences in drug transport, metabolism, and stress adaptation [46].

**Figure 16:**
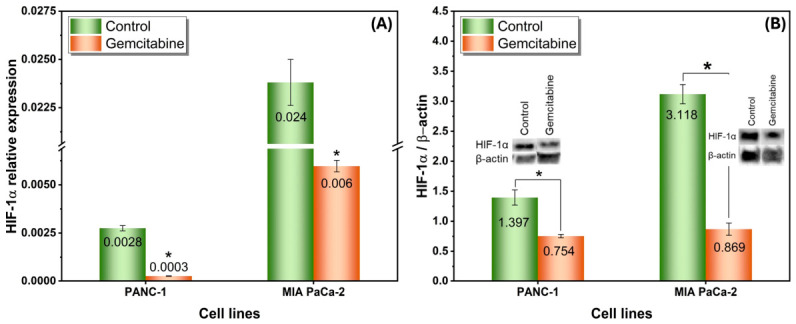
Effect of gemcitabine treatment on HIF-1α expression in 3D pancreatic cancer spheroids: (**A**) Relative HIF-1α mRNA expression levels in PANC-1 and MIA PaCa-2 spheroids cultured in 3% agarose hydrogels under control and gemcitabine-treated conditions, determined by quantitative real-time PCR and normalized to an internal reference gene, and (**B**) Relative HIF-1α protein expression in PANC-1 and MIA PaCa-2 spheroids under the same conditions, assessed by Western blotting and expressed as the HIF-1α/β-actin ratio. Representative immunoblot bands for HIF-1α and β-actin are shown in the insets. Data are presented as mean ± SE. * *p* < 0.05 versus the corresponding control group.

Figure 16 further refines the interpretation of the gemcitabine response by showing that HIF-1α signaling was modulated, but not fully abolished, under treatment conditions. At the transcript level (Figure 16A), gemcitabine reduced HIF-1α expression in both PANC-1 and MIA PaCa-2 spheroids relative to their untreated controls. A comparable trend was observed at the protein level (Figure 16B), where the HIF-1α/β-actin ratio also decreased after treatment in both cell lines. The overall agreement between qPCR and Western blot findings indicates that gemcitabine affected the hypoxia-associated adaptive program at both the mRNA and protein levels. However, HIF-1α expression remained detectable after treatment, and MIA PaCa-2 maintained higher expression than PANC-1 under both control and gemcitabine-exposed conditions, suggesting that this cell line preserved a stronger hypoxic phenotype even during chemotherapy. This observation is biologically meaningful because HIF-1α is a central regulator of glycolytic adaptation, survival signaling, and treatment resistance in oxygen-limited tumor regions [43].

The partial rather than complete suppression of HIF-1α is consistent with the known biology of multicellular tumor spheroids, in which diffusion-limited inner regions continue to provide a hypoxic microenvironment even in the presence of anticancer treatment [16,39]. In such systems, gemcitabine may reduce the proliferative cell fraction and attenuate hypoxia-driven signaling, but it does not necessarily eliminate the structural and metabolic heterogeneity that supports the persistence of tolerant cell populations [39,46]. This interpretation is also consistent with the viability data reported in the same section, where both spheroid models showed reduced but still sustained viability after gemcitabine exposure, and MIA PaCa-2 exhibited a comparatively more tolerant response than PANC-1. The higher residual HIF-1α level observed in MIA PaCa-2 therefore provides a plausible molecular explanation for its more stable viability profile, supporting the view that stronger hypoxia-associated adaptation may contribute to chemotherapy tolerance [43,46]. Taken together, Figure 16 indicates that gemcitabine can attenuate, but not fully reverse, the hypoxia-associated phenotype of pancreatic cancer spheroids. This finding reinforces the physiological relevance of the agarose-based 3D model, as it captures the dynamic interplay between drug exposure and residual hypoxic adaptation that is difficult to reproduce in conventional 2D cultures [16,39,47].

### 2.8. Study Limitations and Future Perspectives

Several limitations of the present study should be acknowledged. First, although quantitative mechanical characterization was incorporated through compressive Young’s modulus measurement, texture profile analysis (TPA), and apparent/effective network density estimation, oscillatory rheology was not performed. Second, direct microstructural characterization of the hydrogel networks, such as SEM imaging, pore-size analysis, or mesh-size estimation, was not available; therefore, the proposed network-level differences should be interpreted as structure–property inferences rather than direct visualization-based confirmation. Third, although HIF-1α was validated at both the mRNA and protein levels, additional hypoxia-associated markers and direct oxygen-mapping approaches were not included, which limits broader mechanistic interpretation of downstream metabolic adaptation. Finally, the conclusions of this study are restricted to the specific GelMA and agarose formulations tested under the present experimental conditions and do not exclude the possibility that alternative GelMA formulations may support different spheroid outcomes. Future studies should therefore combine broader formulation optimization with direct microstructural characterization, expanded hypoxia-marker validation, and additional therapeutic testing to further refine hydrogel selection for pancreatic cancer 3D spheroid models.

## 3. Conclusions

In conclusion, the present findings indicate that hydrogel microenvironment influences the structural and functional relevance of pancreatic cancer 3D models. Although the optimized GelMA formulation used in this study was successfully methacrylated, photocrosslinkable, structurally stable, and cytocompatible, it did not support compact spheroid formation under the tested conditions. The ^1^H-NMR results confirmed a high degree of methacrylation, whereas the mechanical analysis showed that this formulation remained softer and exhibited a lower apparent/effective network density than agarose, suggesting that crosslinking potential alone was not sufficient to generate a microenvironment favorable for spheroid compaction. In contrast, agarose provided a stiffer, non-adhesive, and more mechanically confining network that supported cell–cell aggregation, sustained proliferation, HIF-1α-associated hypoxia signaling, and a physiologically relevant gemcitabine response under the present experimental conditions. These findings should be interpreted as formulation-specific and do not exclude the broader potential of GelMA for spheroid culture after further tuning of stiffness, ligand density, and crosslinking degree. Within the formulation range examined here, agarose appeared to be the more suitable hydrogel platform for pancreatic cancer spheroid modeling and preclinical drug-response studies. Although the present findings support distinct structure–property–function differences between GelMA and agarose, future studies incorporating direct microstructural characterization, such as SEM imaging, pore-size measurement, or mesh-size estimation, will be necessary to validate these network-level interpretations more conclusively. These results nevertheless provide a useful basis for the rational design of hydrogel microenvironments in pancreatic cancer 3D culture systems. Future work should expand this comparative framework by integrating direct network-scale characterization, broader GelMA formulation tuning, additional hypoxia-related markers, and extended therapeutic validation.

## 4. Materials and Methods

### 4.1. Materials

Gelatin (Type A) (G1890, Sigma-Aldrich, St. Louis, MO, USA), methacrylic anhydride, lithium phenyl-2,4,6-trimethylbenzoylphosphinate (LAP), agarose, neutral red, trypan blue, and gemcitabine hydrochloride were purchased from the same supplier. Dulbecco’s Modified Eagle Medium/Nutrient Mixture F-12 (DMEM/F-12), fetal bovine serum (FBS), penicillin/streptomycin, phosphate-buffered saline (PBS), and trypsin-EDTA were obtained from Gibco (Grand Island, NY, USA). Alamar Blue reagent was supplied by Thermo Fisher Scientific (Waltham, MA, USA). All solutions were prepared using sterile distilled water.

### 4.2. Synthesis and Photocrosslinking of GelMA Hydrogels

GelMA was synthesized according to a modified Van Den Bulcke et al. protocol [19]. Briefly, gelatin was dissolved in phosphate-buffered saline (PBS) at 5% or 10% (*w*/*v*) at 60 °C for 30 min. Methacrylic anhydride (MAA, 8% *v*/*v*) was then added dropwise under stirring, and the reaction was maintained at 50 °C for 3 h. The reaction mixture was adjusted to pH 7.4, diluted with an equal volume of PBS, and dialyzed against deionized water (12–14 kDa MWCO) at 37 °C for 7 days, with the water changed twice daily. The purified GelMA solution was sterile-filtered through a 0.22 µm membrane and stored at −20 °C until use.

For hydrogel preparation, GelMA solutions were mixed with lithium phenyl-2,4,6-trimethylbenzoylphosphinate (LAP) at 0.05% or 0.1% (*w*/*v*). Aliquots of 100 µL were transferred into 96-well plates and irradiated at 405 nm with a light intensity of 30 mW/cm^2^ for 0, 15, or 45 s to induce photocrosslinking. After irradiation, PBS was added, and the hydrogels were incubated at 37 °C. Hydrogel stability was evaluated at 24, 48, 72, and 96 h using a semi-quantitative macroscopic scoring system (liquid = 0, partially stable gel = 1, stable gel = 2). The formulation showing the highest structural stability was selected for subsequent biological experiments.

### 4.3. Preparation of Agarose Hydrogels

Agarose hydrogels were prepared at a concentration of 3% (*w*/*v*) by dissolving 0.9 g of agarose in 30 mL of phosphate-buffered saline (PBS). The mixture was heated in a microwave oven until complete dissolution was achieved. The resulting solution was then sterilized by autoclaving at 121 °C for 15 min under standard pressure conditions. After sterilization, the agarose solution was allowed to cool to a workable temperature prior to use.

### 4.4. FTIR and ^1^H NMR Analysis of Hydrogels

Fourier transform infrared (FTIR) spectroscopy was performed to compare the chemical structures of gelatin and synthesized GelMA and to verify the methacrylation of gelatin. Prior to analysis, samples were dried to remove residual moisture. The spectra of gelatin and GelMA were recorded using an ATR-FTIR spectrometer over the range of 4000–600 cm^−1^ with a spectral resolution of 4 cm^−1^ and 32 scans per sample. The obtained spectra were compared in terms of the characteristic amide bands of gelatin and the changes associated with methacrylate functionalization in GelMA.

Proton nuclear magnetic resonance (*^1^H-NMR*) spectroscopy was used to confirm the successful methacrylation of gelatin and to determine the degree of methacrylation (DoM) of GelMA. All measurements were performed using a one-dimensional (1D) *^1^H-NMR* experiment.

Gelatin and GelMA samples were dissolved in deuterium oxide (D_2_O) and analyzed at 50 °C to ensure complete dissolution and to reduce solution viscosity. The spectra were acquired over a chemical shift range of 0–10 ppm. For each sample, 64–128 scans were collected to obtain an adequate signal-to-noise ratio. A relaxation delay (D1) of 5 s was used between scans to allow sufficient longitudinal relaxation. The pulse width was set according to the instrument-specific 90° pulse calibration value. The acquisition time was maintained between 2 and 4 s, and the free induction decay (FID) signals were recorded with 64k data points. Before Fourier transformation, an exponential line broadening factor of 0.5 Hz was applied to improve spectral quality.

All spectra were processed by phase and baseline correction prior to analysis. The degree of methacrylation (*DoM*) of GelMA was calculated from the reduction in the lysine methylene proton signal relative to native gelatin, following a commonly used NMR-based approach for GelMA characterization. Briefly, the integral of the lysine methylene signal at approximately 2.9 ppm in GelMA was compared with that of unmodified gelatin, and the DoM was calculated using Equation (1):

(1) $$DoM(\%)=\left(1-\frac{I_{\mathrm{Lys},\mathrm{GelMA}}}{I_{\mathrm{Lys},\mathrm{Gelatin}}}\right)\times 100$$

where $I_{\mathrm{Lys},\mathrm{GelMA}}$ and $I_{\mathrm{Lys},\mathrm{Gelatin}}$ represent the integrals of the lysine methylene proton peak in GelMA and gelatin, respectively. This method enables quantitative estimation of the extent of methacrylation by measuring the consumption of free amine-containing lysine residues after reaction with methacrylic anhydride.

### 4.5. Swelling and Degradation Assessment

The swelling and degradation behaviors of hydrogels were evaluated under physiological conditions using the optimized GelMA formulation (10% GelMA + 0.05% LAP, 45 s photocrosslinking) and 3% agarose hydrogels. Hydrogel samples were prepared in 96-well plates (100 µL per well) and allowed to stabilize prior to testing.

For swelling measurements, hydrogel samples were air-dried at room temperature (25 °C) for 24 h to obtain the initial weight (*W*_0_). The samples were then immersed in phosphate-buffered saline (PBS) and incubated at 37 °C. At predetermined time points, the hydrogels were removed, gently blotted to eliminate excess surface liquid, and weighed to obtain the swollen weight (*W_t_*). The swelling ratio was calculated using Equation (2) [48]:

(2) $$Swelling\;ratio\;(\%)=\frac{W_{t}-W_{0}}{W_{0}}\times 100$$

This approach provides a relative measure of water uptake based on partially dried initial samples.

For degradation analysis, hydrogel samples were directly incubated in PBS at 37 °C without prior drying. At each time point, samples were collected, gently washed to remove loosely attached residues, and weighed (*W_t_*). The degradation ratio was determined from the percentage of mass loss relative to the initial weight using Equation (3) [37]:

(3) $$Degradation\;ratio\;(\%)=\frac{W_{0}-W_{t}}{W_{0}}\times 100$$

All swelling and degradation experiments were performed in three independent experiments, each conducted with three technical replicates per condition, and the results were expressed as mean ± standard error of the biological replicates. PBS was refreshed periodically to maintain stable experimental conditions.

### 4.6. Mechanical and Textural Characterization of Hydrogels

The mechanical properties of the hydrogels were evaluated by uniaxial compression testing and texture profile analysis (TPA). Compression tests were performed using a SHIMADZU AG-X Plus universal testing machine (Shimadzu Corporation, Kyoto, Japan) to determine the compressive Young’s modulus of the hydrogel samples. For each hydrogel group, five independent samples were tested, and the mean value was calculated. Stress–strain curves were obtained from the compression data, and the compressive Young’s modulus was determined from the slope of the initial linear elastic region. The results were expressed as mean ± standard error (*n* = 5).

In addition, TPA was carried out to assess the textural and viscoelastic properties of the hydrogels, including hardness, adhesiveness, cohesiveness, and springiness. The analysis was performed using a P/3 probe with a trigger force of 25 g and a test speed of 2 mm/s. The parameters obtained from the TPA measurements were used to compare the mechanical behavior and structural integrity of GelMA and agarose hydrogels.

The apparent/effective network density of the hydrogels was further estimated from the compressive Young’s modulus using a modulus-based elastic model. Assuming that the hydrogel network behaved as a nearly incompressible elastic material, the shear modulus (G) was approximated from the compressive Young’s modulus (E) according to G=E/3. The effective network density $\left(\nu_{e,eff}\right)$ was then calculated using Equation (4) [49,50]:

(4) $$\nu_{e,eff}=\frac{G}{RT}=\frac{E}{3RT}$$

where $\nu_{e,eff}$ is the effective network density (mol m^−3^), E is the compressive Young’s modulus (Pa), R is the universal gas constant (8.314 J mol^−1^ K^−1^), and T is the absolute temperature (K). The calculation was performed at 37 °C (310.15 K). The obtained values were used to compare the relative network compactness and mechanical confinement of GelMA and agarose hydrogels under the tested conditions. These values were interpreted as apparent/effective network densities rather than absolute covalent crosslink densities. For agarose, which forms a physically crosslinked network, the calculated values represent an effective junction/network density rather than a true chemical crosslink density.

### 4.7. Cell Culture

Human pancreatic cancer cell lines PANC-1 (ATCC-CRL-1469) and MIA PaCa-2 (ATCC-CRL-1420) were obtained from Istanbul University and Gebze Technical University, respectively. These cells were cultured in DMEM/F-12 medium supplemented with 10% FBS and 1% penicillin/streptomycin. Cells were maintained at 37 °C in a humidified atmosphere containing 5% CO_2_. Cells at 80–90% confluence were detached using trypsin-EDTA and counted before use in the experiment.

### 4.8. Spheroid Formation, Cell Seeding Optimization and Quantitative Morphological Analysis

Sterilized GelMA hydrogels selected for biological experiments and 3% agarose hydrogels were prepared by dispensing 100 µL per well into 96-well plates under aseptic conditions in a biosafety cabinet and allowing them to solidify prior to cell seeding. To evaluate spheroid formation and determine the optimal culture conditions, PANC-1 and MIA PaCa-2 pancreatic cancer cells were trypsinized, counted, and seeded onto the prepared hydrogels at densities ranging from 1000 to 10,000 cells per well. After seeding, the cells were allowed to settle for approximately 5–10 min, followed by the addition of 100 µL of complete culture medium to each well. The plates were then incubated at 37 °C in a humidified atmosphere containing 5% CO_2_. The optimal cell seeding density was selected based on the formation of single, compact, and uniformly shaped spheroids with smooth boundaries and minimal fragmentation. Conditions resulting in irregular aggregates, multiple spheroids per well, or incomplete compaction were excluded. Spheroid formation was monitored at 24, 48, and 72 h using phase-contrast microscopy. Quantitative morphological analysis was performed using ImageJ software (version 1.54g, ImageJ, National Institutes of Health, Bethesda, MD, USA). Spheroid diameter was measured, and circularity was calculated according to the equation 4πA/P^2^, where A is the projected area, and P is the perimeter. Compactness was evaluated using solidity and aspect ratio values. Structural integrity was assessed using a semi-quantitative scoring system based on boundary continuity and aggregation density. All experiments were performed in triplicate (*n* = 3). For ImageJ-based quantitative morphological analysis, at least 7 spheroids per condition were evaluated. Representative images were selected for presentation.

### 4.9. Evaluation of GelMA Cytotoxicity via MEM Elution Assay

The cytotoxicity of photocrosslinked GelMA hydrogels was evaluated using a MEM elution assay in accordance with the guidelines of ISO 10993-5 [36,51]. GelMA samples prepared under optimized conditions were incubated in complete culture medium at 37 °C for 24 h to obtain extraction media containing potential leachables. The extraction was performed using a surface area-to-volume ratio of 3 cm^2^/mL, as recommended by ISO standards for biological evaluation of medical devices [52]. Following incubation, the extracts were sterile-filtered prior to use.

For cytotoxicity assessment, NIH/3T3 fibroblast cells were seeded into 96-well plates at a density of 1000 cells per well and allowed to attach prior to treatment. The conditioned media (GelMA extracts) were then applied to the cells and incubated for 24, 48, and 72 h. Cells cultured in fresh medium served as the negative control, while 2 mM ZnSO_4_ was used as a positive control.

Cell viability was assessed using the Alamar Blue assay. After incubation with the reagent, absorbance values were measured at 570 nm and 600 nm using a microplate reader. The metabolic activity of the cells was calculated by normalizing the absorbance values to the negative control. All experiments were performed in triplicate (*n* = 3).

### 4.10. Viability, Metabolic Activity, and Proliferation Analysis of Spheroids

The viability, metabolic activity, and proliferative status of spheroids were evaluated using trypan blue staining, neutral red staining, and Ki-67 immunohistochemistry. All samples were imaged using optical microscopy, and representative images were selected for qualitative comparison of viability, metabolic activity, and proliferation.

#### 4.10.1. Trypan Blue Staining

Spheroids were incubated with trypan blue solution to assess cell viability based on membrane integrity. Dead cells were stained blue, whereas viable cells excluded the dye. Stained spheroids were imaged using phase-contrast microscopy, and cell viability was qualitatively evaluated.

#### 4.10.2. Neutral Red Staining

Metabolic activity was assessed using neutral red staining, which accumulates in the lysosomes of viable cells. After incubation and washing, spheroids were imaged under a microscope. Viable cells appeared red, which indicated active cellular metabolism.

#### 4.10.3. Ki-67 Immunohistochemical Analysis

To evaluate cellular proliferation, spheroids were fixed, paraffin-embedded, and sectioned using standard histological procedures. Sections were incubated with a Ki-67 primary antibody, followed by detection using an HRP-conjugated secondary antibody and DAB chromogen. Proliferating cells exhibited brown nuclear staining. Due to the structural complexity and heterogeneity of the spheroids, reliable quantitative Ki-67 index analysis could not be performed without introducing substantial segmentation-related bias. Therefore, Ki-67 staining was used as a supportive qualitative indicator to confirm the presence and spatial distribution of proliferating cells within the spheroid structure, rather than as the primary quantitative proliferation endpoint.

### 4.11. Quantitative Real-Time PCR (qPCR) Analysis

Relative HIF-1α expression in PANC-1 and MIA PaCa-2 cells was determined by quantitative real-time PCR (qPCR). Total RNA was extracted using the miRNeasy Mini Kit (Qiagen, Hilden, Germany) according to the manufacturer’s protocol. RNA concentration and purity were assessed spectrophotometrically at 260/280 nm, and only samples with purity ratios between 1.9 and 2.1 were used. RNA integrity was verified by agarose gel electrophoresis. Complementary DNA (cDNA) was synthesized from 1000 ng total RNA per sample using the High-Capacity cDNA Reverse Transcription Kit (Thermo Fisher Scientific, Waltham, MA, USA). qPCR was performed using BlasTaq™ 2X qPCR MasterMix (Applied Biological Materials Inc., ABM, Richmond, BC, Canada) on a CFX96 Real-Time PCR System (Bio-Rad Laboratories, Hercules, CA, USA). The primer sequences for HIF-1α were: forward 5′-TAGCCGAGGAAGAACTATGAACATAA-3′ and reverse 5′-TGAGGTTGGTTACTGTTGGTATCATATA-3′, as previously reported by Qiu et al. (2019) [53]. GAPDH was used as the internal reference gene. Thermal cycling was carried out under manufacturer-recommended conditions, followed by melt curve analysis to confirm amplification specificity. Relative gene expression levels were calculated using the 2^−ΔΔCt^ method described by Livak and Schmittgen (2001) [54]. All qPCR analyses were performed in three independent biological experiments, each analyzed with technical triplicates, and results were expressed as mean ± standard error (SE) of the biological replicates.

### 4.12. Gemcitabine Treatment and Viability Assessment

Preliminary 2D dose–response studies were performed with gemcitabine at concentrations ranging from 1 to 100 mM. The IC50 values were calculated as 17.3 mM for PANC-1 cells and 20 mM for MIA PaCa-2 cells. These concentrations were subsequently applied to the corresponding 3D spheroid models. To assess the chemotherapeutic response, spheroids formed in 3% agarose hydrogels were treated with gemcitabine for 24, 48, 72, and 96 h. After spheroid formation, the culture medium was replaced with fresh medium containing the appropriate gemcitabine concentration, and the plates were incubated under standard culture conditions (37 °C, 5% CO_2_).

Cell viability was assessed using the Alamar Blue (AB) assay, which measures cellular metabolic activity [55]. Briefly, Alamar Blue reagent was added to each well at a final concentration of approximately 10% (*v*/*v*) and incubated at 37 °C. Absorbance values were measured at 570 nm and 600 nm using a microplate reader. Metabolic activity was calculated by normalizing absorbance values to those of untreated control spheroids. Cell viability measurements were obtained from three independent biological experiments, each performed using triplicate wells per condition, and the averaged values from the independent experiments were used for statistical analysis.

### 4.13. Statistical Analysis

For biological assays, each condition was evaluated in three independent biological experiments, and within each biological experiment, measurements were performed using three technical replicates (triplicate wells) unless otherwise stated. Technical replicates were averaged first, and the resulting mean value from each independent experiment was used for statistical analysis; therefore, n refers to the number of independent biological experiments. For quantitative spheroid morphometry shown in Figure 8, at least seven spheroids were analyzed for each condition/time point (*n* ≥ 7). Mechanical compression testing was performed using five independent hydrogel samples per group (*n* = 5). Data were expressed as mean ± standard deviation (SD) or standard error (SE), as indicated in the corresponding figure legends. Statistical analysis was performed using one-way analysis of variance (ANOVA) followed by Tukey’s post hoc test for multiple comparisons. Differences were considered statistically significant at *p* < 0.05. Data processing, graphical representation, and statistical analyses were conducted using OriginPro 2018 (version 2018, OriginLab Corporation, Northampton, MA, USA) and Microsoft Excel (365) (Microsoft Corporation, Redmond, WA, USA).

## Figures and Tables

**Table 1:** Comparative texture profile analysis (TPA) results for GelMA and agarose hydrogels.

Samples	Hardness (N)	Adhesiveness (N·s)	Springiness	Cohesiveness
Agarose	7.01	−1.41	0.99	0.30
GelMA	1.65	0 (Non-adhesive)	1.00	1.00

## Data Availability

Data will be made available by the authors on request.

## Supplementary Materials

The following supporting information can be downloaded at: https://www.mdpi.com/article/10.3390/gels12050377/s1, Figure S1: Aspect ratio of PANC-1 and MIA PaCa-2 spheroids cultured in 3% agarose hydrogels at different time points (24, 48, 72, and 96 h), supporting the morphological characterization presented in the main text.

## Abbreviations

The following abbreviations are used in this manuscript:

GelMA	Gelatin metacryloyl
GEM	Gemcitabine
LAP	Lithium phenyl-2,4,6-trimethylbenzoylphosphinate
PBS	Phosphate-buffered saline
AB	Alamar Blue
DAB	3,3′-Diaminobenzidine
DoM	Degree of Methacrylation
FBS	Fetal Bovine Serum
HIF-1α	Hypoxia-Inducible Factor-1 Alpha
qPCR	Quantitative Real-Time Polymerase Chain Reaction
^1^H-NMR	Proton Nuclear Magnetic Resonance
FT-IR	Fourier Transform Infrared Spectroscopy
PDAC	Pancreatic Ductal Adenocarcinoma
