# Supplementary figures and images for: GelMA Versus Agarose Hydrogels in Pancreatic Cancer 3D Spheroid Modeling: Effects on Morphology, HIF-1α Expression, and Gemcitabine Response
Source: Gels. 2026 Apr 30;12(5):377. doi: 10.3390/gels12050377 (PMC13205332; doi:10.3390/gels12050377)

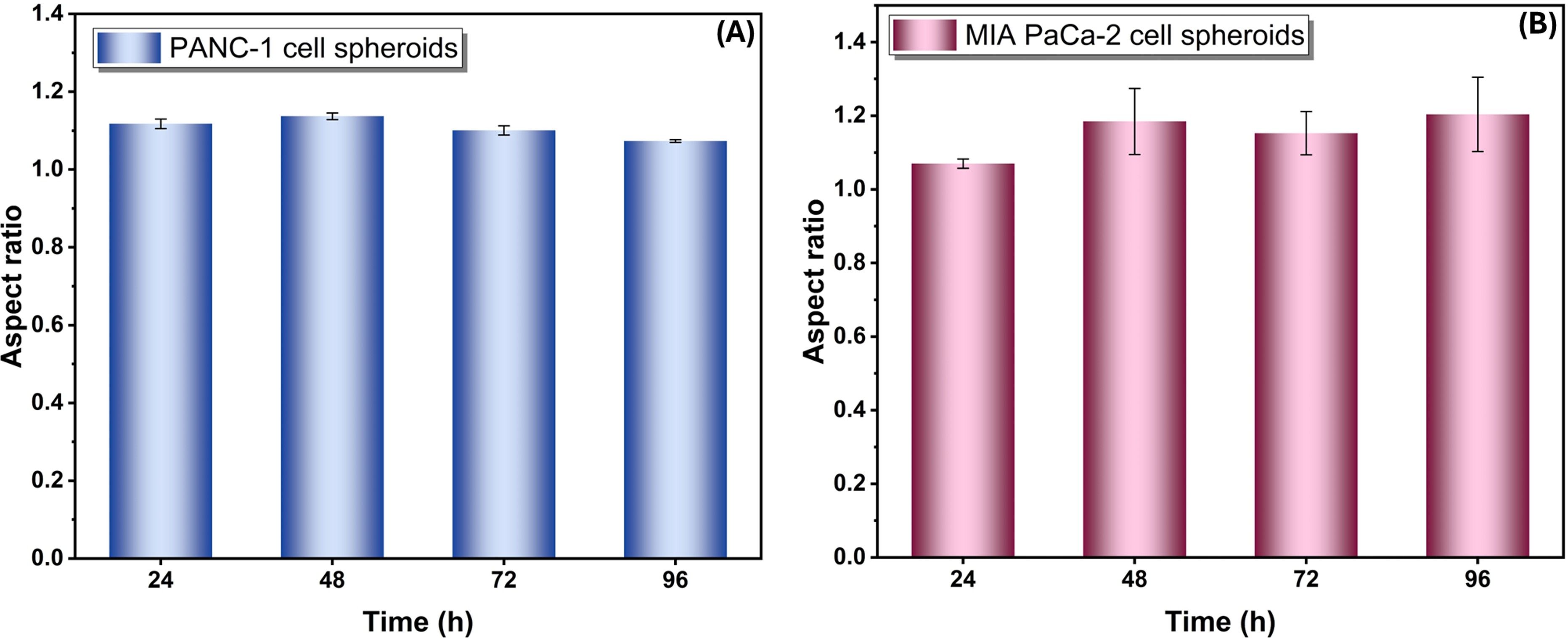

Supplement: Supplementary file 1 [file gels-12-00377-s001.zip › gels-4271963-supplementary.jpg]
